# Bone Strength and Arterial Stiffness Impact on Cardiovascular Mortality in a General Population

**DOI:** 10.1155/2016/7030272

**Published:** 2016-03-07

**Authors:** Petar Avramovski, Maja Avramovska, Aleksandar Sikole

**Affiliations:** ^1^JZU Clinical Hospital “Dr. Trifun Panovski”, Department of Internal Medicine, 7000 Bitola, Macedonia; ^2^University Clinic of Obstetrics and Gynecology, Medical Faculty, “Ss. Cyril and Methodius University” in Skopje, 1000 Skopje, Macedonia; ^3^University Clinic of Nephrology, Medical Faculty, “Ss. Cyril and Methodius University” in Skopje, 1000 Skopje, Macedonia

## Abstract

Osteoporosis and increased arterial stiffness independently have been found to be associated with higher cardiovascular events rates in the general population (GP). We examined 558 patients from GP by dual-energy X-ray absorptiometry (DXA) and pulse wave velocity (PWV) measurements at baseline, with 36-month follow-up period. DXA assessed bone mineral density of femoral neck (BMD FN) and lumbar spine (BMD LS). Carotid-femoral PWV was assessed by pulsed-Doppler. The aim of our study is to find correlation between bone strength and arterial stiffness and their impact on cardiovascular mortality in GP. The mean ± SD of BMD FN, BMD LS, and PWV was 0.852 ± 0.1432 g/cm^2^, 0.934 ± 0.1546 g/cm^2^, and 9.209 ± 1.9815 m/s. In multiple regression analysis we found BMD FN (*β*st = −6.0094, *p* < 0.0001), hypertension (*β*st = 1.7340, *p* < 0.0091), and diabetes (βst = 0.4595, *p* < 0.0046). With Cox-regression analysis, after 17 cardiovascular events, the significant covariates retained by the backward model were BMD FN (*b* = −2.4129, *p* = 0.015) and PWV (*b* = 0.2606, *p* = 0.0318). The cut-off values were PWV = 9.4 m/s, BMD FN = 0.783 g/cm^2^, and BMD LS = 0.992 g/cm^2^. The results for BMD FN and PWV hazard ratio risk were 1.116 and 1.297, respectively. BMD FN as a measure of bone strength and PWV as a measure of arterial stiffness are strong independent predictors of cardiovascular mortality in GP.

## 1. Introduction

As a metabolic disorder, osteoporosis is characterized by low bone mass and bone microarchitectural deterioration [1]. Arteriosclerosis and atherosclerosis as a specific type of arteriosclerosis are phenomena that results with an increase of vascular stiffness and blood flow restriction. Both osteoporosis and atherosclerosis share common risk factors including hypertension, smoking, diabetes, and sedentary lifestyle [2]. Osteoporosis and osteopenia have been found to be associated with higher cardiovascular events rates, arterial wall calcification, and more extensive atherosclerosis [3]. Arteriosclerosis refers to reduced arterial compliance due to increased fibrosis, loss of elasticity, and vessel wall calcification affecting the media of large and middle-sized arteries, with consecutive increase of pulse wave velocity (PWV) as measure of arterial stiffness [4, 5]. Parameters that describe vessel stiffness include compliance and distensibility. The consequence of reduced compliance/distensibility is an increased propagation velocity of the pressure pulse along the arterial tree, called PWV [6]. PWV reflects the elasticity of the segmental artery, stiffer segment of artery results in an increased speed of pulse wave in the artery, which means higher PWV.

These facts accompany the aging process and share common increase of arterial PWV as a measure of vascular stiffness. Increased arterial stiffness independently predicts cardiovascular events in a healthy population. Several studies have examined associations between atherosclerosis at different sites and osteoporosis or low bone mineral density (BMD) in women, and the findings suggest that the development of osteoporosis is a risk for advanced atherosclerosis after menopause (as discussed by Hak et al. 2000 and Sanada et al. 2004) [7, 8].

Association between BMD and vascular stiffness has been the subject of research in several studies (as discussed by Mikumo et al. 2009 [9]; Avramovski et al. 2013 [10]; and Stojanovic et al. 2011 [11]). These latest data support the correlation of atherosclerosis and osteoporosis, indicating the parallel progression of two tissue (bone and vascular) destruction process with increased fatal and nonfatal cardiovascular events, as well as higher fracture risk [9–11]. The progression of these two parallel processes of destruction (BMD and arterial stiffness) was the target of research in our previous studies [12–14]. Arterial stiffening is a marker for increased cardiovascular risk, including myocardial infarction, heart failure, and total mortality, as well as stroke, dementia, and renal disease [15]. Low BMD and bone loss appear to be risk factors for cardiovascular mortality in both women and men [16]. For years, osteoporosis and cardiovascular disease (CVD) were thought to be independent chronic diseases that increased markedly with advancing age. However, increasing evidence now supports a direct association between these chronic conditions [17]. A variety of factors that influence bone metabolism are involved in the development of atherosclerosis and vascular calcification. Interestingly, several bone-related proteins are implicated in the calcification process resulting in mineral deposition [18].

There are limited studies on the association of BMD and arterial stiffness with CVD events rates. Evidence for an association of high PWV, as a measure of arterial stiffness, with low BMD might have implications for screening decision in patients with low bone mass and vice versa. The commonly used imaging modalities to assess bone mass and vascular stiffness use the following imaging technologies of x-radiography and ultrasonography: dual-energy X-ray absorptiometry (DXA) and pulsed-Doppler ultrasound. Data acquired from both the femur and anterior-posterior (AP) spine DXA scans are considered gold standards for diagnosing osteoporosis. Although BMD is considered a reasonable surrogate measure of bone strength, it does not capture aspects of bone geometry that may contribute to fracture risk, such as bone size, shape, and trabecular and cortical properties of bone [19].

Carotid-femoral PWV is considered to be the gold standard for assessing central arterial stiffness [10, 19, 20]. The aim of our study is to assess the correlation between bone strength and arterial stiffness and their impact on cardiovascular mortality in general population.

## 2. Materials and Method

### 2.1. Patients

This study was designed as a longitudinal prospective analysis in 558 patients from the general population (226 men and 332 women) who underwent noninvasive DXA and PWV measurements at the baseline, accompanied by 36-month (35.28 ± 4.13) follow-up period. They had a mean age of 56.24 ± 11.62 years, and their mean body mass index (BMI) was 27.85 ± 4.37 kg/m^2^. One hundred and sixty-four patients were smokers, 61 were insulin-independent diabetes, and 190 were hypertensive. Exclusion criteria were chronic renal disease, insulin-dependent diabetes, degenerative disease (osteoarthritis, chronic obstructive pulmonary disease, Friedreich's ataxia, Marfan's syndrome, etc.), rheumatoid arthritis, liver disease, malignancy, or any other chronic disease that might affect the skeleton or cardiovascular system (stroke, myocardial infarction, and peripheral vascular occlusion).

Demographic and disease history data were collected from the patient's chart and include age, height, weight, history of hypertension, diabetes mellitus, smoking habit, and the diseases mentioned above, which might affect the bone mass and cardiovascular system. Anteroposterior projection of DXA assessed femoral neck BMD and lumbar spine BMD. Carotid-femoral PWV was assessed by pulsed-Doppler ultrasound synchronized with electrocardiography (ECG). Each patient was observed from a baseline during 3 year, for lethal event caused by cardiovascular disease. Cardiovascular mortality was defined as death whose cause was one of the following cardiovascular events or sudden death: cerebrovascular disease (stroke), arrhythmia, peripheral vascular disease, congestive heart failure, or myocardial infarction. All participants signed an informed consent and the Ethics Committee of our institution approved the study.

### 2.2. Assessment

We conducted BMD testing using DXA and arterial stiffness estimation by Doppler sonography in the same day at the beginning of the study (baseline). DXA is enhanced form of X-ray absorptiometry that is used to measure BMD. A DXA scanner is an electronic machine that produces two different X-ray beams, each with different energy levels: low and high. During DXA scan, the soft tissue and the bone will absorb some radiation, and some will travel through the body. Measurement of BMD is based on the difference in absorption of the high and low level X-ray beams across soft and mineral density tissue. Measurement of the BMD is based on the calculated difference of the bone density measurement and of the soft tissue measurement. Doppler ultrasound scanner is a device, which can be used for measurement of blood flow velocities by high frequency probe. We used it to detect the flow signal in carotid and femoral artery to find time delay of blood circulation between these two sites.

### 2.3. BMD

We conducted BMD measuring using DXA scanner QDR4500SL by Hologic (Hologic Inc., Bedford, MA, USA) in two locations: femoral neck and lumbar spine. For assessment of the femoral neck, the patient's foot was placed in a brace that rotates the hip inward. For assessment of the spine, the patient's legs were supported on a padded box to flatten the pelvis and lower the (lumbar) spine. In both cases, the detector was slowly passed over the area generating images on a computer monitor [10, 20]. Patient laid on his back on a flat and open X-ray table. During the scan, which is carried out by radiographer, a large scanning arm with X-ray tube was passed over patient's body to measure bone density in the lumbar spine and hip area. Before we started scanning, we prescanned desired anatomic area to find the best femur neck or lumbar spine position in region of interest (ROI). Small deviations in bone position relative to the scanner were corrected without need to move the patient or mobile console on the bed. From previously done prescan we selected the right area for additional scanning with tool “Global ROI Toolbox.” Scan started in the moment when in “Scan Parameters” appeared in the window, with all parameters of scanned area: Scan Length and Scan Width, Line Spacing, scanned resolution, and energetic beam expressed in kVp (kilovolt peak) and mAs (miliampers seconds). We started the scan by activating a tool “Start Scan” in the same window. After the scan was finished, we selected the icon “Analyze Scan” to get specific data acquired by the previously made scan: area (cm^2^), BMC (bone mineral content), BMD (bone mineral density), *T*-score, and *Z*-score.

Absolute BMD values, *Z*-scores (number of standard deviations [SDs] below an average person of the same age), and *T*-scores (number of SDs below the BMD of a younger reference group) of the lumbar spine and right femoral neck were recorded as BMD (g/cm^2^), *T*-score, and *Z*-score (for femoral neck and lumbar spine from L1 to L4 region). The WHO (World Health Organization) defined the following categories based on bone density in Caucasian females: normal bone, *T*-score greater than −1; osteopenia, *T*-score between −1 and −2.5; osteoporosis, *T*-score less than −2.5 [21].

### 2.4. PWV

We used linear 7.5 MHz probe (Toshiba SSA-340A, Toshiba Medical System Corporation, Tokyo, Japan) at sequential recording of carotid artery and femoral artery. First we recorded the flow wave form of the proximal (carotid artery) and then of the distal site (femoral artery). At both sites, the ECG was also recorded.

**Figure 1:**
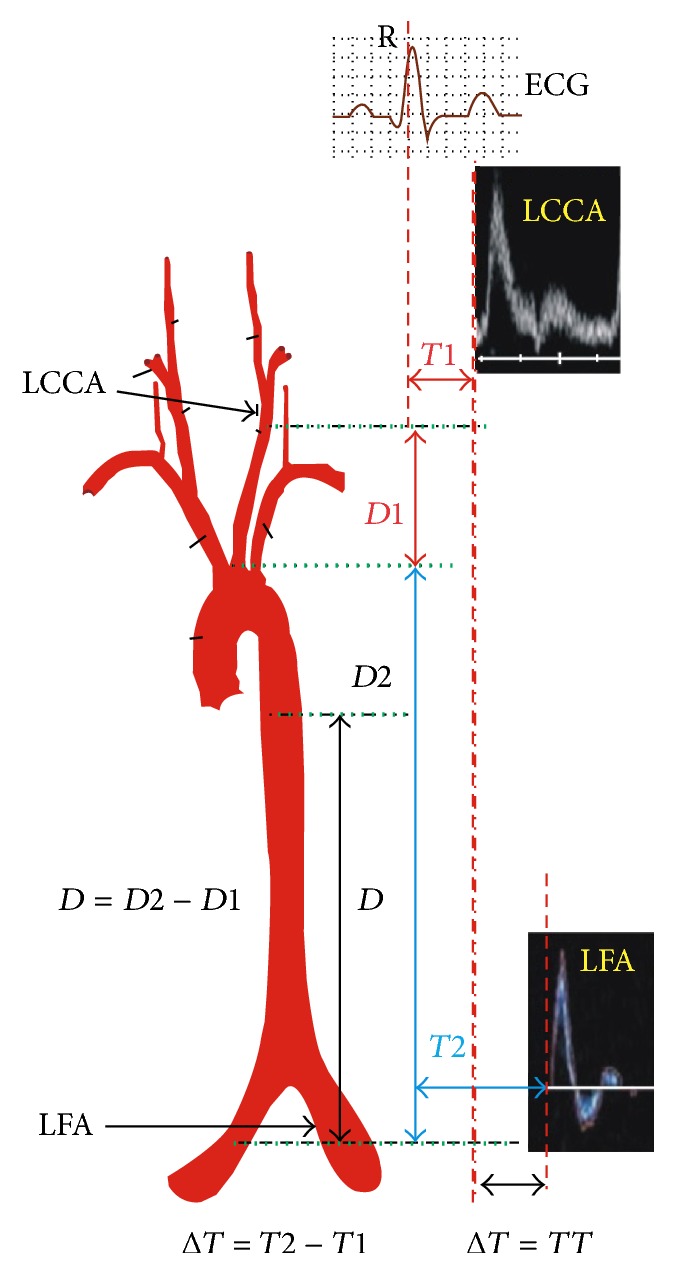
PWV estimation: time diversity of electrocardiographic and carotid-femoral Doppler signal. *TT*(Δ*T*): time delay, LCCA: left common carotid artery, LFA: left femoral artery, and Δ*T*: time diversity.

The proposed method includes the following steps: first step is to acquire flow waveforms by Doppler ultrasound at two locations within left common carotid artery (LCCA) and left femoral artery (LFA) and next step is to detect the delay or the difference in arrival time of the flow wave at the two arterial locations. Distances between the sampling sites *D*  (m) are measured as straight lines between the points on the body surface [10]. PWV measurements were taken in supine position after resting for at least 10 minutes, including a constant room temperature of 19°C to 21°C. Two Doppler waves were recorded transcutaneously at the base of the neck for the LCCA and over the LFA in the groin [22]. The examination began with the patient in a supine position after locating the LCCA with B-mode at the supraclavicular level (1 to 2 cm of the bifurcation). Although it is not possible to analyze the carotid and femoral waves simultaneously, they can be normalized separately with the ECG getting. The same scanning process was repeated on the LFA in the groin. PWV was determined as the “foot to foot” velocity. The distance traveled by the pulse wave was measured over the body surface as the distance between the two recording sites. The distance was assessed using a tape measure located at the same place as the ultrasound probe, with two-dimensional guidance to localize the exact position of the analyzed arterial site [13]. The basic principle of PWV estimation is showed in Figure 1.

We calculated PWV as the ratio of distance (*D*) to transit time (*TT*). The *TT* was determined when we measured the time from R wave of three graphical deflections seen on typical ECG: Q wave, R wave, and S wave, which occur, in rapid succession (QRS) complex to the foot of the waveform using digital measuring calipers. Four to six (dependent of heart frequency) heart measurements were taken and the average was calculated. We acquired the distance (*D*) measured from the sternal notch to the femoral artery at the groin. Dividing the distance “*D*” by time delay “*TT*” we got carotid-femoral PWV [PWV = *D* (meters)/*TT* (seconds)] [23]. The* TT* is the time of travel for the onset of the wave over a known distance and it was estimated by “foot to foot” method. The foot of the wave is defined at the end of diastole, where the rise of the waveform begins.

### 2.5. Statistical Analysis

Statistical analysis was performed using MedCalc for Windows, version 13.0.6.0. (MedCalc Software, Ostend, Belgium). Results are expressed as means ± SD or percentage. Student's *t*-test for paired data was used to compare the results from biomarkers. All tests were two-sided. A value of *p* < 0.05 was considered to indicate statistical significance. Simple linear regression analysis was performed to assess the associations between dependent and independent variables. Bivariate Pearson's correlation analysis was used to find linear relationship between pairs of continuous variables and estimate the strength and direction of their relationships. Multiple linear regression was used to predict outcome of a response variable. Receiver operating characteristics (ROC) curve analysis assessed distinction between patients who survived and patients who died from CV event. Survival rates were analyzed using Kaplan-Meier survival curves. The Cox proportional hazards model was used to identify the independent determinants of mortality predictors.

## 3. Results

### 3.1. Baseline Characteristics of the Patients

During the six-month period from April to September 2014, DXA and carotid to femoral artery PWV measurements and other demographic examinations were successfully conducted on 558 patients from general population aged 56.24 ± 11.62 years (range 37 to 80) and with BMI 27.85 ± 4.37 kg/m^2^ (range 20.76–38.74). The demographic and clinical characteristics of the patients are presented in Table 1.

The mean BMD of the femoral neck was 0.852 ± 0.1432 g/cm^2^, and the mean BMD of the lumbar spine was slightly higher at 0.934 ± 0.1546 g/cm^2^. The mean BMD FN scores were *T*-score = −0.928 ± 1.145 and *Z*-score = 0.085 ± 1.200. The mean BMD spine scores were *T*-score = −1.146 ± 1.415 and *Z*-score = −0.189 ± 1.389.

**Figure 2:**
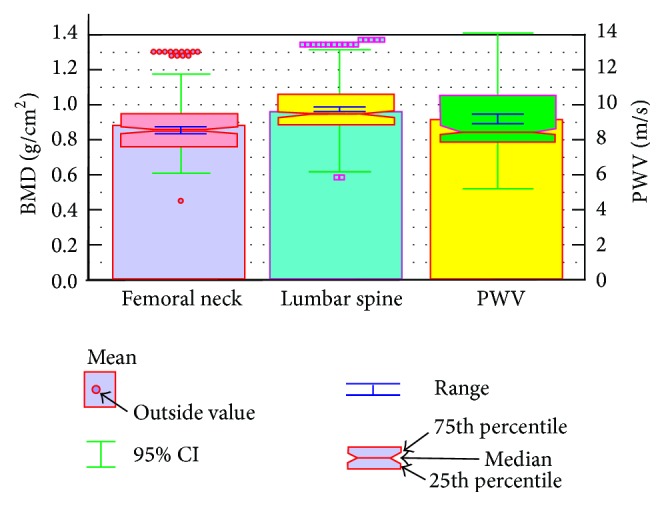
Box plots of the mean, range, median, and 25th and 75th percentiles for tissue biomarkers: femoral neck BMD, lumbar spine BMD, and PWV. Mean, 95% CI of the mean, range, median, 25th and 75th percentiles presents femoral neck BMD and lumbar spine BMD. BMD: bone mineral density; PWV: pulse wave velocity; CI: confidence interval.

The results from paired *t*-test between femoral neck and lumbar spine BMD were as follows: mean difference (0.08272) and two-tailed probability (*p* < 0.0001). The mean PWV velocity was 9.209 ± 1.9815 m/s. The notched box-and-whisker bars for the measured tissue biomarkers of BMD and PWV are presented in Figure 2.

### 3.2. Bivariate Pearson's Correlation Analysis

The positive value of Pearson product-moment correlation coefficient (*r*) as the measure of the strength of linear dependence between two variables [one in the measured tissue markers in the top horizontal row (BMD FN, BMD spine, and PWV) and one in the demographic (age, BMI, hypertension, diabetes, and smokers) or tissue markers (BMD FN, BMD spine, and PWV) in the vertical column] indicated a significant positive correlation between PWV and age (*r* = 0.850, *p* < 0.0001), PWV and hypertension (*r* = 0.306, *p* < 0.0001), PWV and smoking (*r* = 0.188, *p* < 0.0001), BMI and BMD FN (*r* = 0.262, *p* < 0.0001), and BMD FN and BMD spine (*r* = 0.487, *p* < 0.0001). The results from Pearson's correlation are showed in Table 2.

Pearson's correlation revealed a significant inverse correlation between PWV and BMD FN (*r* = −0.327, *p* < 0.0001), PWV and BMD spine (*r* = −0.150, *p* = 0.0004), BMD FN and age (*r* = −0.321, *p* < 0.0001), BMD FN and hypertension (*r* = −0.253, *p* < 0.0001), BMD spine and age (*r* = −0.212, *p* < 0.0001), BMD spine and hypertension (*r* = −0.165, *p* < 0.0001), BMD spine and diabetes (*r* = −0.267, *p* < 0.0001), and BMD spine and smoking (*r* = −0.119, *p* = 0.0048).

### 3.3. Linear Regression Analysis

The results of linear regression, which are an approach for modeling the relationship between a scalar-dependent variable *Y* (PWV) and an explanatory variable, denoted by *X* (BMD FN) were presented as follows: coefficient of determination *R* ^2^ = 0.1150, regression parameter *b*_0_ = 13.267, regression parameter *b*_1_ = 4.729, and equation of simple linear regression *y* = 13.267 − 4.729 · *x*. The coefficient of determination *R* ^2^ (0.1150) showed that only 11.50% of the total variability was explained with the linear relation between PWV and BMD FN or that 11.50% from PWV was dependent on BMD FN. The residual 79.81% of PWV were dependent on other factors, which were not covered with regression model. With each increase of one unit (g/cm^2^) in BMD FN, the PWV score decreased by 4.729. This statement is supported with an accuracy of *p* = 0.0001. Whereas in assessing the linear dependence of PWV from lumbar BMD, we used calculated equation of simple linear regression which showed the average coordination of PWV and lumbar spine BMD variation: *y* = 11.062 − 1.949 · *x*. Using this equation, we obtained the evaluated (theoretical) PWV values compared with its empirical values. The coefficient of determination *R* ^2^ (0.0227) showed that only 2.27% of the total variability was explained with the linear relation between PWV and BMD lumbar spine or that only 2.27% from PWV was dependent on BMD spine (*p* = 0.0004).

### 3.4. Multiple Regression Analysis

We used backward multiple regression analysis to show predictable values of independent variables, also called predictors (age, BMI, BMD FN, spine BMD, hypertension, diabetes, and smoking) on the dependent variable PWV. Assessments [standardized coefficient *β* (*β*st)], standard error of *β*st, *t*, and *p* value on the independent predictor or determinants for increasing of PWV in general population after backward multiple regression analysis are shown in Table 3.

Because of his high collinearity (multicollinearity), meaning that one variable can be linearly predicted from the other variables with a substantial degree of accuracy (variables: age and PWV), age was not included in the model. The *p* values followed the order of statistical significance: BMD FN (<0.0001), hypertension (<0.0091), diabetes (<0.0046), and smoking with nonstatistically significant *p* value 0.0871. There was not statistical significance of *β*st coefficients expressed by *p* value for independent variables: spine BMD and BMI, too. The coefficient of determination *R* ^2^ (0.2899) showed that 28.99% of the total variability was explained with the linear relation between PWV and BMD FN accompanied by other determinants or that 28.99% from PWV was dependent of BMD FN as the predictor and other determinants (hypertension, diabetes, and smoking). There was inverse correlation [negative *β*st (beta standardized) coefficient, *β*st = −6.0094] between the femoral neck BMD and PWV only. This means that any reduction in the femoral neck BMD results in an increased PWV.

### 3.5. Outcomes and Survival Analysis

During the 36-month follow-up period, 17 death from cardiovascular cause were recorded (myocardial infarction 5, stroke 4, arrhythmia 3, congestive heart failure 2, pulmonary edema 2, and ventricular fibrillation 1). According to the Cox-regression analysis, the significant covariates retained by the model (backward stepwise) were only femoral neck BMD and PWV. Assessments (regression coefficient [*b*], hazard ratio coefficient Exp[*b*], *p* value, standard error [SE], and 95% CI [confidence interval] of Exp[*b*]) of independent predictors for cardiovascular outcome after Cox-regression model analysis are shown in Table 4.

Because of the strong intercorrelation of age and PWV (*r* = 0.850; *p* < 0.0001) we did not enter age in Cox-regression analysis. Femoral neck BMD as covariate with negative regression coefficient (*b* = −2.4129) and PWV (*b* = 0.2606) with positive regression coefficient (*b*) are associated with increased hazard and decreased survival times. The predictor PWV has an Exp(*b*) hazard ratio (HR) coefficient of 1.2977 that means the HR increases by 1.2977 (29.77%) with each unit increase in PWV. Femoral neck BMD indicate HR increase by 11.16071 (1/0.0896) with each unit decrease by self, or 1.116071 with each 0.1 g/cm^2^ decreases of femoral neck BMD.

### 3.6. Estimation of Cut-Off Point

**Figure 3:**
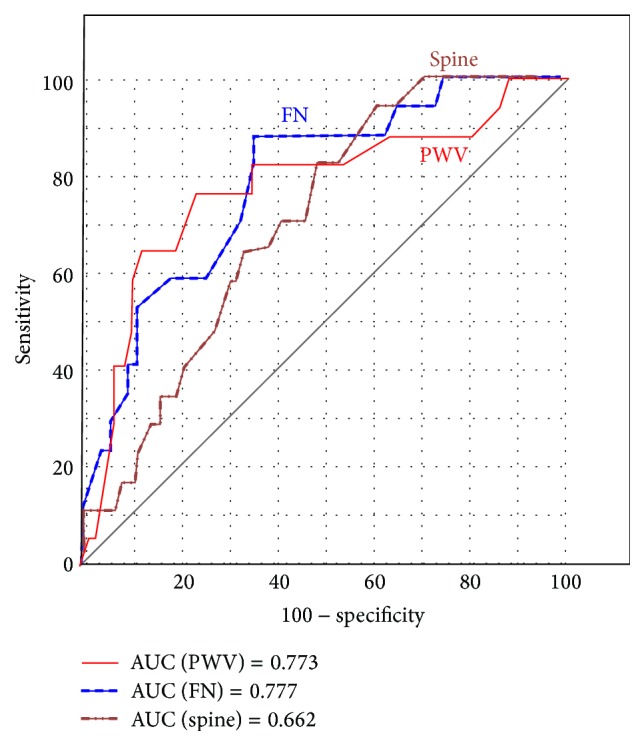
Receiver operating characteristics curves for PWV, BMD of femoral neck and lumbar spine as a prognostic marker for cardiovascular event. PWV: pulse wave velocity; FN: femur neck; AUC: area under the ROC curve (AUC).

We used discrimination, the ability of a model (estimation of cut-off point) to distinguish between patients with or without cardiovascular event, exactly, patients who survived and who died. We assessed them by receiver operating characteristic (ROC) curve analysis, a fundamental statistic tool for diagnostic test evaluation. The summary image of three ROC curves for PWV, BMD FN, and BMD spine as a prognostic marker for cardiovascular event is shown in Figure 3.

Each point on the ROC curves (PWV, BMD FN, or BMD spine) represents a sensitivity/specificity pair corresponding to a particular decision threshold (PWV, BMD, or BMD spine in detection of cardiovascular mortality): PWV (sensitivity 80.9% and specificity 64,7%), BMD FN (sensitivity 88.2% and specificity 63,8%), and BMD spine (sensitivity 82.4% and specificity 48,6%). The cut-off values, which correspond to the respective values for sensitivity/specificity pair, were PWV = 9.4 m/s, BMD FN = 0.783 g/cm^2^, and BMD spine = 0.992 g/cm^2^, and they represent the highest sensitivity and specificity pair for appropriate tissue biomarker in the detection of cardiovascular mortality. The ROC curves are shown by different color and line styles (red solid line, filled brown dash-dot line, and filled blue dashed line). The greater the value of AUC coefficient, the greater the surface of the curve above the diagonal and the greater the predictor value for cardiovascular death. The results of pair wise comparison of three ROC curves showed no statistical significance (*p* = 0.9669) between PWV and BMD FN ROC curves, but there was statistical significance (*p* = 0.0346) between PWV and BMD spine ROC curves.

### 3.7. Kaplan-Meier Survival

**Figure 4:**
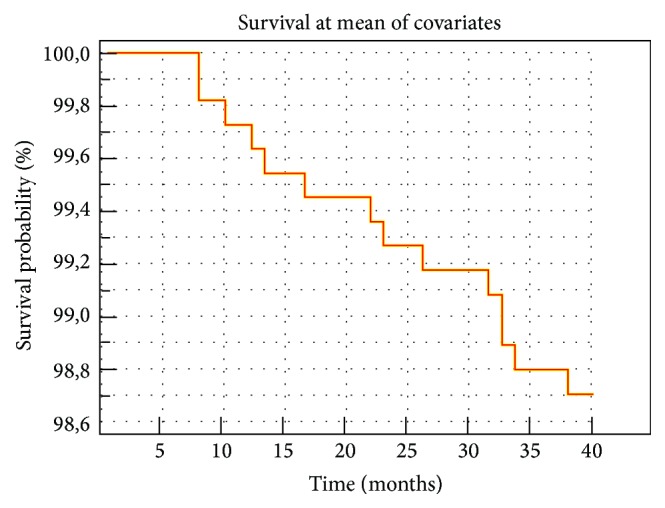
Kaplan-Meier estimates of cardiovascular survival in general population.

A plot of the Kaplan-Meier estimate of the survival function presented as series of horizontal steps of declining magnitude approaching the true survival function in general population patients is shown in Figure 4.

## 4. Discussion

In this prospective longitudinal study with 36-month follow-up period, we studied a cohort of 558 patients from the general population with mean age of 56.24 ± 11.62 years. Each participant was subjected to two different noninvasive diagnostic methods: DXA and Doppler ultrasonography. The purpose of this study was to find correlation between bone strength and arterial stiffness and their impact on cardiovascular mortality in the general population.

Among all osteoporotic fractures, hip fractures are the location most commonly associated with mortality. Literature in the past two decades showed that most deaths are related to associate comorbidities, reflecting a poor underlying health condition rather than the fracture itself [24]. BMD have shown to be associated with mortality independently of age, weight, BMI, smoking status, previous fracture, physical activity, drug use, and presence of chronic disease [25]. There are lots of numbers of studies that examine the overall mortality in patients with low bone mass, unlike a small number of studies that examined cardiovascular mortality in the same patients. We can rightly point out that our study is the first and unique because it examines the impact of PWV and BMD in predicting cardiovascular risk and survival in patients from the general population. The researched group did not include a young healthy population; it consisted of participants from the general population who were not spared from the normal process of atherosclerosis, aging, and osteoporosis.

We found that lumbar spine BMD (0.934 ± 0.1546 g/cm^2^) was greater than FN BMD (0.852 ± 0.1432 g/cm^2^) and this difference was statistically significant (*p* < 0.0001) calculated by paired *t*-test. This difference (ΔBMD) comes from the fact that DXA relied on measurement of the relative absorption of dual-energy X-ray beams blindly projected through the body. The dense aortic calcification absorbs more quantum of X-ray beam than the spine, causing a falsely elevated BMD reading [26]. This falsely elevated BMD spine result is most pronounced in elderly. The evidence of calculated ΔBMD (BMD spine minus BMD FM) in the population younger than 55 years is smaller (0.0613 ± 0.1589 g/cm^2^) than the ΔBMD in older (>55 years) population (0.105 ± 0.1373 g/cm^2^). This distinction certainly stems from the growing amount of calcification in the aorta that leads to a corresponding increase of aortic stiffness and PWV, consequently for each of the age groups (<55, 7.960 ± 1.1631 m/s; >55, 10.801 ± 1.6371 m/s).

We found significant (*p* < 0.0001) inverse correlation of PWV and BMD FN (*r* = −0.327) and PWV and BMD spine (*r* = −0.150) by bivariate Pearson's correlation. That means that there is a significant correlation between the two processes, decreasing hip and vertebral bone density measured by DXA and increasing vascular stiffness as measured by carotid-femoral PWV.

The vascular calcification that occurs in elderly and osteoporotic patients is likely responsible for a substantial increase in vascular stiffness as measured by PWV [27]. In the adynamic state in osteoporosis, excessive bone resorption causes rapid accrual and removal of calcium and phosphorus from the bone. Poor bone mineralization and excessive calcium and phosphorus in the circulation likely favor deposition of hydroxyapatite crystals in soft tissues [28]. We found associative a connection of diabetes with BMD. The fact that the study included relatively small number of diabetic's participants with type 2 diabetes (10.93%) was the reason that strong and statistically significant associative connection between PWV and diabetes could not be shown. That is why, the multiply regression which is made will provide that correlation. The mechanism of bone loss in diabetes is still unknown. Insulin-like growth factors and other cytokines may influence diabetic bone metabolism [29]. In this study, there is an inverse correlation between bone strength (both for BMD FN and BMD spine) and hypertension also and between bone strength and PWV. Hypertension is presumed to be relate to alteration in calcium metabolism leading to increased calcium losses, secondary activation of the parathyroid gland, and increased movement of calcium from bone [30]. Increased angiotensin II levels in hypertensive patients have a harmful effect by increasing bone resorption and inhibiting mineralization [31]. The presence of hypertension is an independent predictor of low bone density, as discussed by Yazici et al. 2011 [32].

In our study, by linear regression we found inverse close correlation between PWV as a scalar-dependent variable and BMD FN as an explanatory variable. Because of the negative value of *b*_1_ coefficient (−4.729) in linear regression equation, we discuss that, with each increase of one unit (g/cm^2^) in BMD FN, the PWV value decreases 4.729 times. Kim et al. 2014 discussed [33] that the correlation between BMD and vascular stiffness has not been clearly established in epidemiology and clinical studies. The pathophysiology for the correlation between BMD and vascular stiffness is also not clear. Age, smoking, hypertension, diabetes, hyperlipidemia, renal failure, physical activity, and menopause are the risk factors, and factors like inflammatory factor, oxidative LDL, osteopontin, vitamin D, and estrogen are predicted to have an effect on the correlation between BMD and vascular stiffness and osteoprotegerin has been highlighted as an important factor [34].

In multiple regression analysis, we found an independent predictor with inverse correlation (BMD FN, *p* < 0.0001) for arterial stiffening expressed by PWV (Table 3). The identification of a high-risk subset of population by DXA will be an important element of effective preventive strategies for bone resorption and atherosclerosis. Detection of low BMD by DXA will set indication for diagnostic PWV estimation of arterial stiffness. By multiple regression analysis, we found diabetes and hypertension which increased PWV, as determinants with inverse correlation with PWV. BMD FN plays superior role as independent predictor over other variables (spine BMD, Age, BMI, hypertension, diabetes, and smoking) because of his relative high coefficient of determination *R* ^2^ (0.2899). That means one-third of PWV changes is dependent on BMD FN as the predictor only, versus two-thirds which belong to other determinants, above-mentioned variables.

By Cox-regression analysis we detected the predictor of cardiovascular mortality. PWV increases the HR risk for 1.2977 times (29.77%) with each unit increase in PWV. Several mechanisms may explain the association between increased PWV and cardiovascular mortality. Arterial stiffness is a cause of premature return of reflected waves in late systole, increasing central pulse pressure and the load on the ventricle, reducing ejection fraction, and increasing myocardial oxygen demand. Arterial stiffness is correlated with atherosclerosis probably through the effects of cyclic stress on arterial wall thickening [35]. Aortic PWV may represent a surrogate end point, which may in fact indicate which patients with osteoporosis and the traditional cardiovascular risk factors translate into real risk. Our finding (HR = 1.297) is close to summary comparative results from meta-analysis of the predictive value of PWV for cardiovascular events presented by Vlachopoulos et al. 2010 [36]: HR = 1.6 [37], HR = 1.44 [38], and HR = 1.20 [39]. Importantly, the predictive value of increased arterial stiffness is larger of the patients with higher risk disease states, such as osteoporosis or renal disease [40]. The principal finding of our study was that joint effects of PWV and BMD FN were strongly and independently predictive of outcome in the general population patients. The femoral neck BMD showed superior predictable value for cardiovascular events than PWV by Cox-regression analysis (*p* = 0.015 versus *p* = 0.0318) in our study. The BMD FN shown in ROC curves analysis superior sensitivity (88.2% versus 80.9%) and greater surface of the curve above the diagonal (0.777 versus 0.773 and 0.662, Figure 3) emphasizes stronger diagnostic value of BMD FN in predicting cardiovascular event than of PWV and BMD spine. Taking into consideration the above-mentioned arguments, it remains to be demonstrated whether BMD FN, which is a major determinant of bone strength, have any independent prognostic relevance for cardiovascular events. Due to the small statistical difference of BMD and PWV as independent predictors (*p* = 0.9669), their role in cardiovascular risk predicting became equal.

The appropriate cut-off values for PWV and BMD FN represent their highest sensitivity and specificity pair in detection of cardiovascular mortality. Cardiovascular disease and osteoporosis as common age-related condition traditionally were considered unrelated and their coexistence was attributed to independent age-related process. However, an increasing body of biological and epidemiological evidence has provided support for a link between the two conditions that cannot be explained by age alone [41]. One of the main reasons for the increase in vascular stiffness is calcification of arteries. Atherosclerotic calcification and bone mineralization share a number of intriguing common features. Calcification of the arterial tissue is not merely a passive process of calcium phosphate precipitation or adsorption in end-stage atherosclerosis, but instead it is a highly organized process that is regulated by mechanisms similar to those involved in bone mineralization [42]. Osteoporosis and cardiovascular disease shared similar etiological factors (such as smoking, physical activity, alcohol intake, menopause, hypertension, etc.), which may simultaneously promote or inhibit atherosclerosis and bone demineralization and could partly explain the association between the two diseases [43]. Low BMD and bone loss appear to be risk factors for cardiovascular mortality at both male and female. The Study of Osteoporotic Fractures (SOF) showed that an increase in BMD loss at the hip in the order of one standard deviation (SD) was associated with a 1.3-fold increase in cardiovascular mortality among white female 65 years of age and older [16]. The results of our study are very close to the results of above-mentioned studies: femoral neck BMD indicate HR increase by 1.116 with each unit decrease of density raised over his cut-off value (0.783 g/cm^2^). A plot of Kaplan-Meier shows the survival functions approaching the true survival function in general patients at mean of covariates. Evidently 70.58% (12/17) of the patients who died have BMD femoral neck below cut-off point (0.783 g/cm^2^) and 64.70% (11/17) of the patients who died have PWV greater than cut-off point (9.4 m/s). The tendency to hold this values of bone density and PWV below the critical values (0.783 g/cm^2^ and 9.4 m/s) leads to a significant reduction of cardiovascular risk.

The salient finding of our study was that BMD FN and PWV were strong-independent predictors of cardiovascular mortality with high-level performance values, assessed by simple, indirect, reproducible, and noninvasive evaluation of regional arterial stiffness and bone mineral density. In conclusion, the current study's findings suggest that BMD FN as measure of bone strength and PWV as a measure of arterial stiffness are strong independent predictors of cardiovascular mortality in general population patients. In our study, high degree of correlation between BMD and PWV suggests that the process of atherosclerosis has many common pathophysiological and epidemiological factors with the process of osteoporosis.

A standard PWV cut-off point in the general population should be further investigated and estimated by large clinical studies. By incorporating DXA BMD and carotid-femoral Doppler PWV measurements into standard regular diagnostic assessments for cardiovascular estimation, the patients which are at increased cardiovascular risk can be pinpointed earlier, with a recommendation for preventive appropriate osteopenia treatment and stiffness reduction by therapy starting.

## Figures and Tables

**Table 1:** Demographic characteristic of the patients.

Characteristics	Mean SD, *n* (%)	Range
Age, years	56.24 ± 11.62	37–80
Height, cm	166.97 ± 10.08	150–193
Weight, kg	77.88 ± 15.31	50–120
BMI, kg/m^2^	27.85 ± 4.37	20.76–38.74
Hypertension	190 (34.05)	/
Diabetes	61 (10.93)	/
Smokers	164 (29.39%)	/

Values are presented as mean ± SD or number (%). BMI: body mass index.

**Table 2:** Bivariate Pearson's correlation analysis of demographic characteristic with BMD and PWV.

*n* = 558	BMD FN, g/cm^2^		BMD spine, g/cm^2^		PWV, m/s	
	*r*	*p*	*r*	*p*	*r*	*p*
Age, years	−0.321	<0.0001	−0.212	<0.0001	0.850	<0.0001
BMI, kg/m^2^	0.262	<0.0001	0.038	0.375	0.065	0.124
Hypertension	−0.253	<0.0001	−0.165	<0.0001	0.306	<0.0001
Diabetes	−0.165	<0.0001	−0.267	<0.0001	0.076	0.074
Smokers	−0.053	0.213	−0.119	0.0048	0.188	<0.0001
BMD FN, g/cm^2^	/	/	0.487	<0.0001	−0.327	<0.0001
BMD spine, g/cm^2^	0.487	<0.0001	/	/	−0.150	0.0004
PWV, m/s	−0.327	<0.0001	−0.150	0.0004	/	/

The results of the bivariate Pearson's correlation analysis of demographic characteristic with BMD and PWV are presented as (*r*) indexes and (*p*) values. BMD: bone mineral density; BMI: body mass index; FN: femoral neck; PWV: pulse wave velocity.

**Table 3:** Multiple backward regression analysis of determinants of PWV.

Multiple regression			Dependent *Y*, PWV	
Sample size (558)				
Coefficient of determination *R* ^2^		0.2899		
Residual standard deviation		1.6923		
Multiple correlation coefficient		0.5385		

Regression equation				

Independent variables	Coefficient *β*st	Std. error	*t*	*p*

BMD FN, g/cm^2^	−6.0094	0.5402	−11.124	<0.0001
Hypertension	1.7340	0.1607	10.793	0.0091
Diabetes	0.4595	0.1617	2.842	0.0046
Smoking	0.3341	0.1949	1.714	0.0871

Variables not included in the model: spine BMD, age, and BMI.

BMD: bone mineral density; BMI: body mass index; FN: femoral neck; PWV: pulse wave velocity;  *β*st: beta standardized coefficient; Std. error: standard error.

**Table 4:** Cox-regression survival analysis (predictors of cardiovascular outcome).

Cox proportional-hazards regression						
Cases summary						

Number of events	17	3.05%				
Number censored	541	96.95%				
Total number of cases	558	100.00%				
Overall model fit			Method Backward			
Null model −2Log likelihood			Enter variable if *p* < 0.05			
Full model −2Log likelihood			Remove variable if *p* > 0.1			
Chi-squared	22.384					
DF	2					
Significance level						

Coefficients and standard errors						

Covariate	*b*	SE	Wald	*p*	Exp(*b*)	95% CI of Exp(*b*)

Femoral neck BMD	−2.4129	1.6975	2.0205	0.015	0.0896	0.033 to 0.4011
PWV	0.2606	0.1214	4.6109	0.0318	1.2977	1.0242 to 2.4527
Variables not included in the model						
Spine BMD						
